# Loop mediated isothermal amplification of *Clostridioides difficile* isolates in gastrointestinal patients

**DOI:** 10.1186/s13568-022-01382-1

**Published:** 2022-04-12

**Authors:** Mojtaba Moosavian, Razieyeh Keshavarzi, Effat Abbasi Montazeri, Eskandar Hajiani

**Affiliations:** 1 Infectious and Tropical Diseases Research Center, Health Research Institue, Ahvaz Jundishapur University of Medical Sciences, Ahvaz, Iran; 2 Department of Microbiology, Faculty of Medicine, Ahvaz Jundishapur University of Medical Sciences, Ahvaz, Iran; 3 Division of Gastroenterology and Hepatology, Department of Internal Medicine, Ahvaz Jundishapur University of Medical Sciences, Ahvaz, Iran

**Keywords:** LAMP assay, PCR, Culture, *Clostridioides difficile*, *tcdA*, *tcdB*

## Abstract

This study investigated the prevalence of *Clostridioides difficile* by culture, multiplex polymerase chain reaction (M-PCR), and loop mediated isothermal amplification (LAMP) in patients with suspected *C. difficile* infections (CDIs). Also, the results of three methods were compared. All stool specimens collected from CDI suspected patients were cultured on selective *C. difficile* cycloserine-cefoxitin fructose agar and incubated in an anaerobic jar up to 7 days. The bacterial isolates were identified using standard tests. Multiplex-PCR (M-PCR) was performed for detection of *tcdA*, *tcdB*, and *tpi* genes. The LAMP assay was performed to detect the *tcdB* gene of *C. difficile*. *C. difficile* was isolated from 20.0% (n = 10/50) of samples by culture. M-PCR showed that 34.0% (n = 17/50) of the specimens were positive for *C. difficile* based on the presence of *tpi* gene. Out of the 17 *C. difficile*, 13 strains (76.0%) were positive for *tcdB* gene using M-PCR. However, the LAMP assay showed that 30.0% (15/50) of specimens were positive for the presence of *tcdB* gene. M-PCR and LAMP methods showed 100.0% sensitivity compared to the culture method. However, the specificity of the LAMP (87.5%) was relatively higher than the M-PCR (82.5%) compared to the culture. Based on the results of this study, the prevalence of toxigenic *C. difficile* strains was high in suspected CDI patients. So, the differentiation between toxigenic and non-toxigenic strains is necessary. Our data showed that the LAMP assay is a good method for direct detection of toxigenic *C. difficile* strains from stool specimens.

## Introduction

*Clostridioides difficile* is a Gram-positive bacillus, strictly anaerobic, spore-forming, and toxin-producing bacterium that exists in the soil and the gastrointestinal tract of animals and humans (Czepiel et al. 2019). For the first time in 1978, this bacterium was considered a significant contributor to antibiotic-associated diarrhea. This bacterium can cause many infections including limited mild diarrhea, pseudomembranous enterocolitis, toxic megacolon, sepsis, bowel obstruction, and abdominal perforation (Bagdasarian et al. 2015; Chen et al. 2017).

The main risk factors of *C. difficile* infections (CDIs) are the use of broad-spectrum antibiotics, especially in patients with impaired immune systems, cancer, and burns, as well as the use of proton pump inhibitors (PPIs). Elderly people and hospitalized patients are also more susceptible to this pathogen (Zhou et al. 2019; Leffler and Lamont 2015).

Centers for Disease Control and Prevention (CDC) has announced the *C. difficile* as a serious threat for hospitalized patients requiring immediate attention and control. The incidence of CDI in hospitalized patients increases hospitalization costs and mortality (Marra et al. 2020). The pathogenicity of this bacterium is dependent on the production of two main toxins, enterotoxin A (TcdA) and cytotoxin B (TcdB), which induces cell death and inflammatory reactions in the intestine (Burnham and Carroll 2013). These toxins can be detected for diagnostic purposes using cell culture assays (toxin B) and different enzyme immunoassays (EIAs) (Burnham and Carroll 2013). Diagnosis of *C. difficile* is usually based on the clinical history and several laboratory tests including the cell culture cytotoxin B assay (CTBA) that remained the reference standard for detection of cytotoxin-producing *C. difficile* and culture of cytotoxin-producing *C. difficile* isolates (TC). However, rapid toxin A and B enzyme immunoassays, frequently used to save cost and labor, often display suboptimal sensitivity and are no longer recommended. Today, molecular methods including polymerase chain reaction (PCR) and Real-time PCR are commercially available for the detection of *C. difficile* (Usacheva et al. 2016; Lee et al. 2021).

In recent years, a novel rapid loop-mediated isothermal amplification (LAMP) technique is being developed to diagnose infectious diseases (Yu et al. 2017). This high-speed and high-specificity method uses six types of primers to detect eight regions of DNA. This method also uses the polymerase enzyme which has field-shifting activity and can detect six specific sequences in the DNA. Perhaps the best advantage of the LAMP method is that it does not require a thermocycler to be performed. Instead, this method can be done on any device that can create a temperature range of 63–65 °C such as water bath (Keikha 2018). Therefore, these characteristics led to the use of the LAMP test as a suitable method for the detection of *C. difficile* in hospitals (Yu et al. 2017; Doing and Hintz 2012). Although the CDI has been reported to increase rapidly from Europe and North America, little information is available from the Middle East including Iran (Goudarzi et al. 2013).

Since there have been very few studies in the southwestern region of Iran to identify this bacterium by different methods, this study aimed to investigate the prevalence of *C. difficile* by three methods: culture, Multiplex-PCR (M-PCR), and LAMP in patients with suspected CDIs. Also, the efficacy of M-PCR and LAMP methods in detection of *C. difficile* were compared to the culture method as the reference standard method. Finally, the LAMP method was compared with the M-PCR in the detection of toxigenic *C. difficile*.

## Materials and methods

### Ethics approval

This study was settled and performed according to the Declaration of Helsinki and obtained approval from the Research Ethics Committee (REC) of the Ahvaz Jundishapur University of Medical Sciences, Ahvaz, Iran (Code No: IR.AJUMS.REC.1396.444). All patients provided written informed consent.

### Specimen’s collection

Hospitalized CDI suspected patients who were referred to Emam Khomeini, Razi, and Abuzar educational hospitals affiliated to Jundishapur University of Medical Sciences Ahvaz, Khuzestan Province, southwest of Iran from July 2017 to May 2018 (11 months), were enrolled in this study. The CDI suspected patients were selected by a gastroenterologist physician who was resident in the hospitals and based on clinical findings and laboratory tests. All stool samples were collected from the selected patients and transferred to the Department of Microbiology of Ahvaz Jundishapur University of Medical Sciences, during 1–4 h.

### Processing and culture of samples

All stool specimens were treated with alcohol to inhibit non-sporulating organisms and enhance the isolation of *C. difficile*. In brief, about 1 g of each stool sample was added to an equal volume of 95% ethanol (Merck, Germany) and 1 mL of the Brain heart infusion (BHI) broth medium (Biolife, Italia). Then, all specimens were slowly vortexed and incubated at room temperature for 45–60 min (Goudarzi et al. 2013; Bailey and Scott’s 2015). The treated stool suspensions were cultured on selective *C. difficile* cycloserine- cefoxitin fructose agar (CCFA, Sigma, USA) containing supplement (D-cycloserine 250 mg; cefoxitin 8 mg) and 7% defibrinated sheep blood. Plates were incubated in an anaerobic jar (United Kingdom) (containing 10% H_2_, 10% CO_2_, 80% N_2_) at 37 ˚C for 48 h. Negative cultures were incubated for up to 7 days.

Bacterial isolates were presumptively identified as *C. difficile* by characteristic morphology of colony with gray color (2–3 mm in diameter), specific horse-stable odor, and Gram-positive appearance (Bailey and Scott’s 2015; Zarandi et al. 2017). Finally, all phenotypically identified *C. difficile* isolates were stored in brain heart infusion (BHI; Merck, Germany) containing 20% glycerol at − 70 ˚C for further molecular identification.

### DNA extraction

Genomic DNA was extracted from pure colonies of *C. difficile* isolates using the boiling method as described previously (Legaria et al. 2018; Abbasi Montazeri et al. 2020). Also, DNA was extracted directly from stool specimens using the QIAamp DNA Stool Minikit kit (QIAGEN, Hiden, Germany), according to the manufacturer's recommendations. The concentration and purity of extracted DNA were measured using a NanoDrop spectrophotometry (Thermo Scientific, Waltham, MA, USA) at 260 nm (Legaria et al. 2018; Abbasi Montazeri et al. 2020).

### Multiplex-PCR assay

Multiplex-PCR (M-PCR) was carried out for detection of toxin *Clostridioides difficile* A (*tcdA*), toxin *Clostridioides difficile* B (*tcdB*), and triose phosphate isomerase (*tpi*) genes using specific primers (Table 1) (Persson et al. 2008, Silva et al. 2011). The specificity of the primers was checked using primer-BLAST tools (https://www.ncbi.nlm.nih.gov/tools/primer-blast/) (Ye et al. 2012). The final PCR reactions were done in a total volume of 25 μl. The reaction mixture contained 1 × buffer (10 mM Tris–HCl, 50 mM KCl), 1.5 mM MgCl_2_, 0.2 μM of each deoxynucleoside triphosphate (dNTP), 0.3 μM of each primer, 1.25 U of Taq DNA polymerase enzyme and 20 ng of DNA as a template. M-PCR programs were done by Eppendorf thermocycler (Roche Co., Germany) as follows: one cycle initial denaturation at 94 °C for 3 min followed by 35 cycles of denaturation for 1 min at 95 °C, annealing for 30 s at 55 °C, extension at 72 °C for 1 min and final extension of 72 °C for 5 min. The *C*. *difficile* strain ATCC 9689 was used as a positive control for three genes.

**Table 1:** The sequences of special primers for the *tcdA, tcdB* and *tpi* genes

Target gene	Primer sequence (5′-3′)	Size of product (bp)	Reference
*tcdA-F* *tcdA-R*	F:GCATGATAAGGCAACTTCAGTGGTA R:AGTTCCTCCTGCTCCATCAAATG	629	Persson et al. 2008
*tcdB-F* *tcdB-R*	F:GAGCTGCTTCAATTGGAGAGA R:GTAACCTACTTTCATAACACCAG	412	Persson et al. 2008
*tpi-F* *tpi-R*	F:AAAGAAGCTACTAAGGGTACAAA R:CATAATATTGGGTCTATTCCTAC	210	Silva et al. 2011

### PCR products electrophoresis

The PCR products were analyzed using electrophoresis on a 1.5% agarose gel containing 0.5 μg/ml safe stain (Sinaclon Co., Tehran, Iran) under 80 V for 40 min. The bands were visualized under UV light using a gel documentation device (Protein Simple, Santa Clara, CA, USA).

### Primer design for LAMP assay

The LAMP assay was performed with 3 primer pairs that was specific for *tcdB* gene of *C. difficile* according to previously described study (Table 2) (Kato et al. 2005). The used primers included two inner primers, forward inner primer (FIP) and backward inner primer (BIP), two outer primers, F3 and B3 and two loop primers: loop forward (LF) and loop backward (LB). The efficiency of the primers used in the LAMP method was evaluated and confirmed using basic local alignment search tool (BLAST) software on the NCBI server (http://blast.ncbi.nlm.nih.gov/Blast.cgi). The primers were commercially produced by takapouzist Company (Tehran, Iran).

**Table 2:** The sequences of special primers for the LAMP assays based on *tcdB* gene

*TcdB* gene	Sequence (5′-3′)	Reference
Outer primers	F3: GTATCAACTGCATTAGATGAAAC B3: CCAAAGATGAAGTAATGATTGC	Kato et al 2005
Inner primers	FIP: CTGCACCTAAACTTACACCATCTATCCTTCCTACATTATCTGAAGGATT BIP: GAGCTAAGTGAAACGAGTGACCCGCTGTTGTTAAATTTACTGCC	
Loop primers	LB: AATAGTTGCAATTATAGG LF: AGACAAGAAATAGAAGGCTAGG	

### LAMP assay

The LAMP assay was performed in a total 25 μl reaction mixture containing 3 μl dNTP (1.4 mM), 3 μl betaine (0.8 M) (Sigma, USA), 2 μl MgSO_4_ (4 mM) (New England Biolabs, USA), 2.5 μl enzyme bst2 warm start (New England Biolabs), 2.5 μl isothermal amplification buffer (1X) containing Tris–HCl (20 mM), Kcl (10 mM), (NH4)_2_SO_4_ (10 mM), MgSO_4_ (4 mM) (New England Biolabs, USA), 0.5 μl of each B_3_ and F_3_ primers (20 PM), 2 μl of each primers FIP and BIP (40 PM) and 1 μl of each LF and LB loop primers (20 PM). The sequences of specific LAMP primers have been shown in Table 2. The reaction mixture was placed into a hot block plate (Boeco, Germany) at 60 °C for 1 h, followed by incubation at 80 °C for 10 min to terminate the Bst DNA-polymerase activity. The *C*. *difficile* strain ATCC 9689 and distilled water were used as a positive control and negative control, respectively.

### Detection of LAMP products

For the detection of LAMP products, 1 μl of SYBR Green-I (Thermo-Fisher Scientific, USA) 0.1% was added to each sample and observed under UV light laminator (Protein Simple, Santa Clara, CA, USA). The results showed positive tubes in green and negative tubes in orange. For confirmation of the results, the electrophoresis of the LAMP products also was performed on 2% agarose gel with DNA safe stain for 60 min (Moosavian et al. 2019).

### Statistical analysis

The results were entered into the SPSS version 16 software (IBM Armonk, NY, USA) and statistical analysis was carried out using appropriate tests such as chi-square. In this test, *P*-value was considered statistically significant ≤ 0.05 (Ejikeugwu et al. 2019; Sheikh et al. 2020). For LAMP and M-PCR, the specificity, sensitivity, positive predictive value (PPV), and negative predictive value (NPV) were calculated and compared to the culture results. Likewise, the LAMP was compared with the M-PCR in the detection of the toxigenic *C. difficile*. Cohen's kappa (κ) was calculated to assess the agreement of LAMP and M-PCR with the culture. A κ correlation value of 0.40 or below indicates weak agreement, 0.41–0.60 indicates good agreement, and above 0.60 indicates strong agreement (Phetsuksiri et al. 2020).

## Results

### Detection of *C. difficile* by culture method

Totally, 50 stool samples were collected from CDI patients who were hospitalized in gastrointestinal, infectious, transplant, and emergency wards. Out of these patients, 28 (56%) were female and 22 (44%) were male, respectively. The average age of patients was 32.8 years. Demographic information of patients are presented in Table 3.

**Table 3:** Demographic information of studied patient

Methods	Culture positive	PCR positive	*tcdB *PCR positive	*tcdB* LAMP positive
Number				
Gender				
Male (22)	4 (4/22)	7 (7/22)	6 (6/22)	6 (6/22)
Female (28)	6 (6/28)	10 (10/28)	7 (7/28)	9 (9/28)
Antibiotic				
Consumption (31)	9 (9/31)	16 (16/31)	13 (13/31)	14 (14/31)
No - consumption (19	1(1/19)	1 (1/19)	0 (0/19)	1 (1/19)
Age (year)				
1–20 (10)	1 (1/10)	1 (1/10)	0 (0/10)	1 (0/10)
21–30 (12)	1 (1/12)	4 (3/12)	2 (2/12)	2 (3/12)
31–40 (11)	1 (1/11)	2 (2/11)	2 (2/11)	2 (2/11)
41–50 (8)	3 (3/8)	5 (5/8)	4 (4/8)	5 (5/8)
≤ 50 (9)	4( 4/9)	5 (5/9)	5 (4/9)	5 (5/9)
Hospitalization time				
< 1week (13)	1 (2/13)	2 (2/13)	1 (3/13)	1 (1/13)
1–2 week (22)	4 (4/22)	5 (5/22)	4 (4/22)	4 (4/22)
> 2week (15)	5 (3/12)	10 (10/12)	8 (8/12)	10 (10/12)
Hospital departments				
Gastrointestinal (19)	4 (4/19)	7 (7/19)	7 (7/19)	7 (7/19)
Infectious (20)	5 (5/20)	6 (6/20)	5 (5/20)	5 (5/20)
Transplant (6)	1 (1/6)	3 (3/6)	1 (1/6)	3 (3/6)
Emergency (5)	0 (0/5)	1 (1/5)	0 (0/5)	0 (0/5)

The results of this study led to the isolation of 10 (20%) *Clostridioides* species from 50 non-repetitive stool samples by culture method. The grown colonies on the selective medium were identified based on Gram and spore staining and their morphology such as transparent gray, with a diameter of 2–3 mm, obtuse end, and the smell of horse stable contained subterminal spore whose diameter was usually larger than a bacterium. All 10 isolates were positive for the *tpi* gene using M-PCR and were confirmed as *C. difficile*.

### Detection of *tpi, tcdA and tcdB* genes by M-PCR assay

**Fig. 1:**
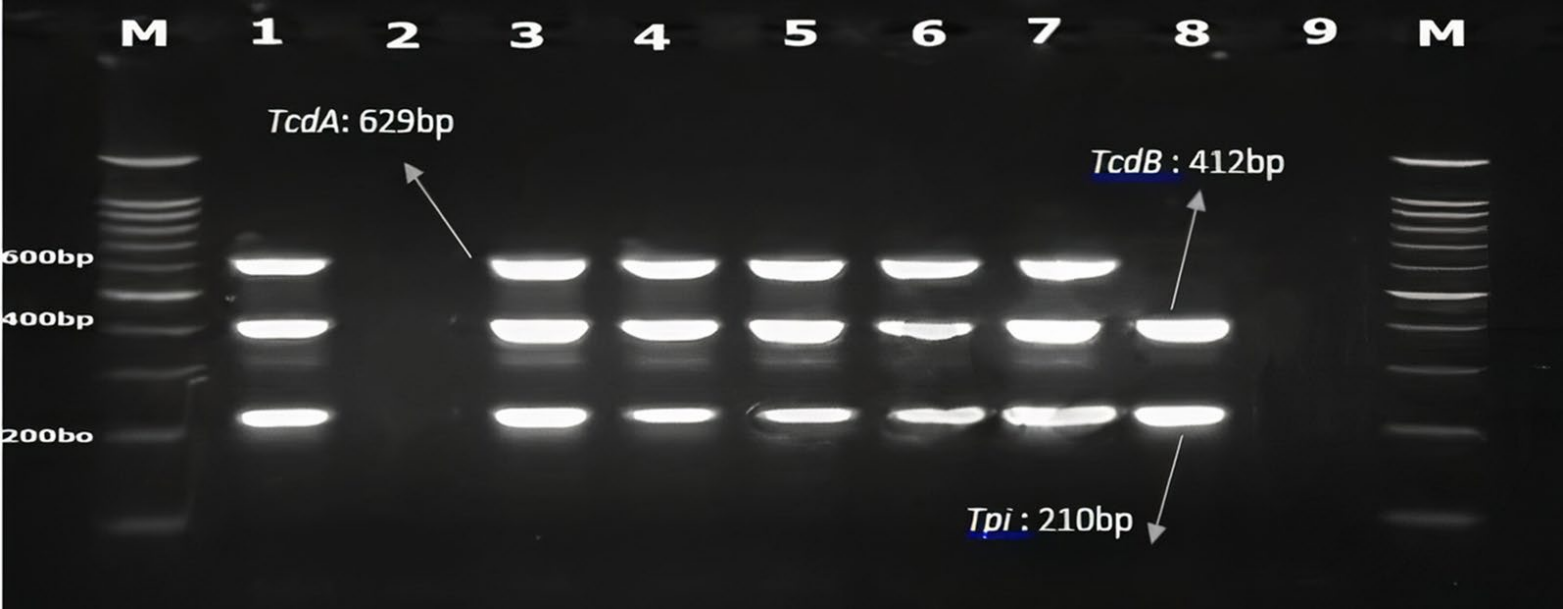
Multiplex-PCR for *tpi*. *tcdA* and *tcdB* genes. Lane 1: Control positive: *Clostridioides difficile* ATCC9689; Lane 2: Control negative: distilled water; Lane 3–7: patiens sample TcdA + TcdB + ; Lane 8: patients sample TcdA- TcdB + ; M: DNA ladder 100 bp

Results of M-PCR revealed that 34% (n = 17/50) of stool specimens were positive for the presence of the *tpi* gene of *C. difficile*. In total, of the 17 confirmed strains, 13 (76.5%) were toxigenic, from which 12 strains (70.6%) were positive for *tcdA* and *tcdB* genes (*tcdA*^+^*tcdB*^+^). Also, one strain (5.9%) was positive only for toxin B (*tcdA*^*−*^*tcdB*^+^) (Fig. 1). All of these patients had taken antibiotics. Most of the antibiotics used by these patients included penicillins, fluoroquinolones, and cephalosporins. Also, four strains (23.5%) had no toxin gene. Also, all samples that showed positive growth in the culture method had positive M-PCR result.

### LAMP assay for *tcdB* gene

**Fig. 2:**
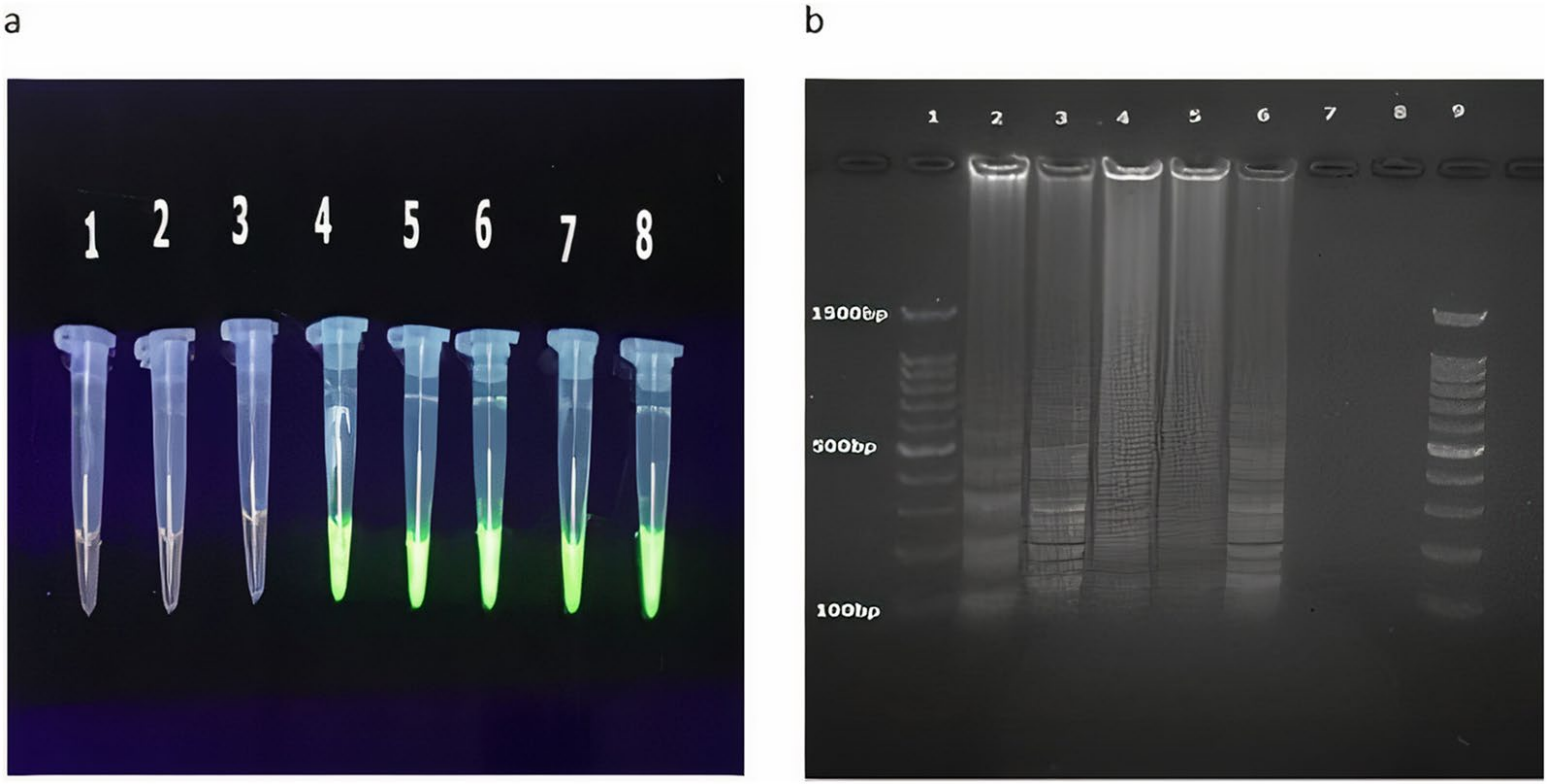
Loop-mediated isothermal amplification of *ctdB* products: **a** SYBR Green-I results of loop-mediated isothermal amplification of *ctdB* products. Lane 1: control negative; Lane 2 and 3: negative patient samples; Lane 4–7: positive patient samples; Lane 8: Control positive: *Clostridioides difficile* ATCC9689; **b** Electrophoresis results of loop-mediated isothermal amplification of *ctdB* products. Lane 1 and 9: DNA ladder 100 bp; Lane 2: Control positive: *Clostridioides difficile* ATCC9689; Lane 3–6: Positive patient samples; Lanes 7 and 8: negative controls

The results of LAMP assay showed that 30% (n = 15/50), of specimens were positive for toxigenic *C. difficile* strains based on the detection of the *tcdB* gene (Fig. 2). The results of visual observation and electrophoresis of these strains were quite similar (Fig. 2). The highest percentage of bacterial isolates was related to the infectious ward and the lowest was related to the emergency ward (Table 3). Also, all samples that showed positive growth in the culture method had positive LAMP result. Likewise, all samples that were *tcdB* gene positive with M-PCR, showed positive results in LAMP assay.

### Comparison of M-PCR and LAMP with culture method

The efficacy of the M-PCR and LAMP assays in comparison to the culture method is shown in Table 4. Both methods showed 100.0% sensitivity compared to the culture method. However, the specificity of the LAMP (87.5%) was relatively higher than the M-PCR (82.5%) compared to the culture. In comparison to the culture, the test accuracy rates of LAMP and M-PCR were 90.0% and 86.0%, respectively. Also, κ coefficient showed a good agreement (0.7) of the M-PCR and LAMP assays with the culture method.

**Table 4:** Test performance of the multiplex polymerase chain reaction (M-PCR) and loop mediated isothermal amplification (LAMP) in detection of *Clostridioides difficile* isolates compared to the culture method

	Culture for *Clostridioides difficile*		
	Positive	Negative	Total
M-PCR Positive	10 (20.0%)	7 (14.0%)	17 (34.0%)
M-PCR Negative	0 (0.0%)	33 (66.0%)	33 (6.0%)
Total	10 (20.0%)	40 (80.0%)	50 (100.0%)
Sensitivity (%) (95% CI)	100.0% (69.2 to 100.0%)		
Specificity (%) (95% CI)	82.5% (67.2 to 92.7%)		
Positive predictive value (%) (95% CI)	58.8% (42.2 to 73.7%)		
Negative predictive value (%) (95% CI)	100.0%		
Test accuracy (%) (95% CI)	86.0% (73.3 to 94.2%)		
Kappa coefficient (κ)	0.7		
LAMP Positive	10 (20.0%)	5 (10.0%)	15 (30.0%)
LAMP Negative	0 (0.0%)	35 (70.0%)	35 (70.0%)
Total	10 (20.0%)	40 (80.0%)	50 (100.0%)
Sensitivity (%) (95% CI)	100.0% (69.2 to 100.0%)		
Specificity (%) (95% CI)	87.5% (73.2 to 95.8%)		
Positive predictive value (%) (95% CI)	66.7% (46.8 to 82.0%)		
Negative predictive value (%) (95% CI)	100.0%		
Test accuracy (%) (95% CI)	90.0% (78.2 to 96.7%)		
Kappa coefficient (κ)	0.7		

### Comparison of the LAMP assay with the M-PCR in detection of the toxigenic *C. difficile*

The comparison of the LAMP with the M-PCR in the detection of the toxigenic *C. difficile* is shown in Table 5. The sensitivity, specificity, and accuracy rates of LAMP in detection of toxigenic *C. difficile* in comparison with the M-PCR method were 100%, 94.6%, and 96.0%, respectively. Also, κ coefficient showed a good agreement (0.9) of the LAMP with the M-PCR method. The LAMP method detected more *tcdB* positive *C. difficile* (30.0%, n = 15/50) than M-PCR assay (26.0%, n = 13/50). However, the difference between the two methods was not statistically significant (*P*-value = 0.15).

**Table 5:** Comparison of the loop mediated isothermal amplification (LAMP) with the multiplex polymerase chain reaction (M-PCR) in the detection of the toxigenic *Clostridioides difficile*

	M-PCR for toxigenic *Clostridioides difficile*		
	Positive	Negative	Total
LAMP Positive	13 (26.0%)	2 (4.0%)	15 (30.0%)
LAMP Negative	0 (0.0%)	35 (70.0%)	35 (70.0%)
Total	13 (26.0%)	37 (74.0%)	50 (100.0%)
Sensitivity (%) (95% CI)	100.0% (75.3 to 100.0%)		
Specificity (%) (95% CI)	94.6% (81.8 to 99.3%)		
Positive predictive value (%) (95% CI)	86.7% (62.8 to 96.2%)		
Negative predictive value (%) (95% CI)	100.0%		
Test accuracy (%) (95% CI)	96.0% (86.3 to 99.5%)		
Kappa coefficient (κ)	0.9		

## Discussion

Today, the old methods for detecting the toxigenic *C. difficile*, which are mostly based on culture and immunoassay, do not have enough sensitivity and efficiency. Therefore, most researchers in more recent studies have turned to molecular methods (Xiao et al. 2020).

In the present study, three methods were used for the detection of *C. difficile* in suspected patients with hospitalized CDI. The result of the culture method showed the 20.0% (n = 10/50) frequency rate for *C. difficile*. Using culture method, in previous studies by Shoaei et al. (2019) from Isfahan, Iran and Baghani et al. (2020) from Tehran, Iran, a higher (28.6%) and a lower (8.8%) frequency rate was reported for *C. difficile*, respectively. Also, Putsathit et al. (2017) from Thailand and Plants-Paris et al (2019) from Kenya reported the *C. difficile* strains in 23.7% (n = 100/422) and 37.6% of the stool samples using the direct culture method. Differences in the results of various studies can be due to differences in the quality and type of culture medium used, differences in the age of the study population, differences in customs and health habits of the geographical studied area, and sampling methods.

In this study, we used the CCFA as a selective medium for the isolation of *C. difficile* from fecal specimens. In different studies, various culture media have been used to detect *C. difficile* (Shoaei et al. 2019; Putsathit et al. 2017; Plants-Paris et al. 2019). Today, with the help of newer specific culture media such as ChromID agar, the detection of bacteria by phenotypic methods has become better and more efficient. In this regard, Putsathit et al. (2017) from Thailand and Zhou et al. (2019) from China, used the ChromID agar culture medium to detect *C. difficile* in their studies. However, one of the disadvantages of phenotypic methods such as culture is their inability to differentiate between toxin-producing and non-toxin-producing strains of *C. difficile* (Chung et al. 2019). Therefore, the use of molecular methods such as PCR, real-time PCR, and LAMP assay is more sensitive and specific options in this regard and can detect the toxigenic *C. difficile* strain in different sources (Zhou et al. 2019; Marcos et al. 2021).

In this study, the M-PCR was another method to evaluate the prevalence of *C. difficile* in the patient population. The findings revealed that 34.0% (n = 17/50) of the fecal samples were positive for the *tpi* specific gene of *C. difficile* that was higher than the results obtained by the culture method. In another study by Shoaei et al. (2019) from Iran, the direct detection of *tpi* gene in fecal samples was reported equal to 28.6% that was lower than this study. Various studies have used different genes to detect this pathogen in stool samples of humans and animals. One of these genes is *16S rRNA* which was used in Alimolaei et al. (2019) and Samir et al. (2021) studies. Using this gene, the aforesaid researchers reported a prevalence of 90% and 43.7% for *C. difficile* from Egypt and Iran, respectively. In this study, the M-PCR could detect 7 *C. difficile* in culture negative samples and showed a sensitivity, specificity, and test accuracy of 100.0%, 82.5%, and 86.0% compared to the culture method. Also, this test had a good agreement (κ coefficient = 0.7) with the culture method. Hence, M-PCR can be used instead of time-consuming culture method to detect the *C. difficile* more quickly. Another advantage of M-PCR is the simultaneous detection of toxigenic *C. difficile*, which is not possible with the culture method. In a previous study by Lai et al. (2018), multiplex polymerase chain reaction (PCR) coupled with capillary electrophoresis (mPCR-CE) compared with the BD MAX Cdiff and real-time cell analysis assay (RTCA) in detection of toxigenic *C. difficile*. The results revealed that mPCR-CE had a specificity of 97.2% and a sensitivity of 96.0%, which was lower than BD MAX Cdiff but higher than RTCA. Another study by Ahn et al. (2020) showed that M-PCR could detect noticeably more diarrhea causing pathogens including *C. difficile* is stool samples than culture method.

Another notable finding of this study was the high prevalence rate of 70.6% (n = 12/17) for t*cdA* + *tcdB* + *C. difficile* strains identified by M-PCR. Also, one strain (5.9%) was *tcdA*^*−*^* tcdB*^+^ and 4 (23.5%) strains were *tcdA*^−^ *tcdB*^−^. In line with our results, the previous reports from different countries have shown the higher prevalence of *tcdA* + *tcdB* + *C. difficile* strains compared to *tcdA- tcdB* + isolates (Zhou et al. 2019; Putsathit et al. 2017). Recent studies have shown the clinical importance of *C. difficile* strains that produce only toxin B (*tcdA*^*−*^* tcdB*^+^) (Goudarzi et al. 2013). The prevalence of this strain has been reported 38% and 22% in Asia and Africa, respectively (Putsathit et al. 2017; Plants-Paris et al. 2019). In this study, a total of 23.5% of isolates had no toxin genes. In the previous report by Zarandi et al. (2017) from Iran, a higher proportion of non-toxigenic (48.9%) strains has been detected. In contrast to our finding, the Plants-Paris et al. (2019) from Kenya has reported a lower prevalence (10.2%) of non-toxigenic *C. difficile* strains. The high frequency of non-toxigenic *C. difficile* colonization may play a significant role in lowering the risk of developing CDI. This is because patients with non-toxigenic *C. difficile* colonization may have a strong enough immune response to *C. difficile* toxins (Zainul et al. 2017).

Although PCR is a sensitive method for the detection of *C. difficile*, it requires expensive devices that are not available in many clinical laboratories. The LAMP assay is a simpler and faster molecular method for detection of several bacteria that does not require expensive equipment for performing in clinical diagnostic laboratories. Thus, in recent years, some companies have produced commercially available diagnostic LAMP kites for different pathogens (Obande and Banga Singh 2020). In this research, we used the LAMP assay for the detection of *tcdB* gene using previously introduced primers (Kato et al. 2005). The results showed that LAMP method detected more *tcdB* positive *C. difficile* (30.0%, n = 15/50) than M-PCR assay (26.0%, n = 13/50). However, the difference between the two methods was not statistically significant (*P*-value = 0.15). LAMP method showed a sensitivity, specificity, and test accuracy of 100.0%, 87.5%, and 90.0% compared to the culture method. Also, LAMP assay had a good agreement (κ coefficient = 0.7) with the culture method. When compared to the M-PCR, the LAMP showed a sensitivity, specificity, and test accuracy of 100.0%, 94.6%, and 96.0%. Also, this test had a good agreement (κ coefficient = 0.9) with the M-PCR. LAMP could detect 5 *C. difficile* in culture negative samples. Positive samples by LAMP but negative by culture were all positive by M-PCR, and so we concluded that there were no false-positives. In line with the current findings, a meta-analysis by Wei et al. (2015) revealed that LAMP method had a pooled sensitivity and specificity of 93.0% and 98.0% in detection of *C. difficile* in stool samples. In a study by Yu et al. (2017) from China, the LAMP method showed a tenfold more sensitivity than PCR in the detection of *cdtA* and *cdtB* toxin genes. In another experiment by McElgunn et al. (2014) from the United States, a new method based on the LAMP technique for the detection of *C. difficile* was investigated. It was found that this method has a sensitivity of 95% and a specificity of 100% compared to the gold standard cytotoxigenic culture method in detecting the bacterium. Also, Wang et al. (2019) found that the LAMP assay is a reliable procedure for the detection of *C. difficile tcdA* and *tcdB* toxin genes in feces of critically ill patients. One of the limitation of this study was the small sample size. It is recommended to repeat the current study with greater sample size to approve the better performance of this method compared to other existing assays. Based on the high frequency of toxigenic *C. difficile* strains in our region, accurate and quick diagnostic testing for these bacteria is critical for patient care and the prompt adoption of infection control measures. To the best of our knowledge, the current research is the first study in Iran that used the LAMP method for detection of toxigenic *C. difficile*. Also, there were rare studies on the diagnosis of this bacterium by LAMP assay.

In conclusion, based on the results of this study, the prevalence of toxigenic *C. difficile* strains was high in suspected CDI patients, especially in hospitalized patients in southwest Iran. So, the differentiation between toxigenic and non-toxigenic strains is necessary. Our data showed that the LAMP assay is a good method for direct detection of toxigenic *C. difficile* strains from stool specimens and does not require expensive devices. Also, this method is comparable to the M-PCR in detection of toxigenic *C. difficile*. Therefore, this method is recommended for clinical laboratories that do not perform routine molecular tests and in laboratories that do not have the purchasing power of expensive molecular devices.

## Data Availability

All analyzed data within this study can be obtained from the corresponding author on request.

## Abbreviations

BHI: Brain heart infusion
CTBA: Cell culture cytotoxin B assay
CDC: Centers for Disease Control and Prevention
CDI: *Clostridioides difficile* Infection
EIA: Enzyme immunoassay
LAMP: Loop mediated isothermal amplification
M-PCR: Multiplex polymerase chain reaction
